# Transcranial Ultrasonic Focusing by a Phased Array Based on Micro-CT Images

**DOI:** 10.3390/s23249702

**Published:** 2023-12-08

**Authors:** Yuxin Yin, Shouguo Yan, Juan Huang, Bixing Zhang

**Affiliations:** 1 Institute of Acoustics, Chinese Academy of Sciences, Beijing 100190, China; yinyuxin@mail.ioa.ac.cn (Y.Y.); yanshouguo@mail.ioa.ac.cn (S.Y.); zhbx@mail.ioa.ac.cn (B.Z.); 2 University of Chinese Academy of Sciences, Beijing 100049, China

**Keywords:** transcranial ultrasound focusing, pulse compression, phased array, micro-computed tomography

## Abstract

In this paper, we utilize micro-computed tomography (micro-CT) to obtain micro-CT images with a resolution of 60 μm and establish a micro-CT model based on the k-wave toolbox, which can visualize the microstructures in trabecular bone, including pores and bone layers. The transcranial ultrasound phased array focusing field characteristics in the micro-CT model are investigated. The ultrasonic waves are multiply scattered in skull and time delays calculations from the transducer to the focusing point are difficult. For this reason, we adopt the pulse compression method and the linear frequency modulation Barker code to compute the time delay and implement phased array focusing in the micro-CT model. It is shown by the simulation results that ultrasonic loss is mainly caused by scattering from the microstructures of the trabecular bone. The ratio of main and side lobes of the cross-correlation calculation is improved by 5.53 dB using the pulse compression method. The focusing quality and the calculation accuracy of time delay are improved. Meanwhile, the beamwidth at the focal point and the sound pressure amplitude decrease with the increase in the signal frequency. Focusing at different depths indicates that the beamwidth broadens with the increase in the focusing depth, and beam deflection focusing maintains good consistency in the focusing effect at a distance of 9 mm from the focal point. This indicates that the phased-array method has good focusing results and focus tunability in deep cranial brain. In addition, the sound pressure at the focal point can be increased by 8.2% through amplitude regulation, thereby enhancing focusing efficiency. The preliminary experiment verification is conducted with an ex vivo skull. It is shown by the experimental results that the phased array focusing method using pulse compression to calculate the time delay can significantly improve the sound field focusing effect and is a very effective transcranial ultrasound focusing method.

## 1. Introduction

Transcranial ultrasonic focusing is a noninvasive energy focusing method and has a variety of application scenarios in brain neuromodulation and tumor therapy. Currently, transcranial ultrasonic focusing is efficacious in the noninvasive treatment of malignant gliomas [1,2,3], neuromodulation of the function of specific brain regions [4,5], and neurodegenerative diseases such as Alzheimer’s disease and Parkinson’s disease [3,6,7]. However, due to the inhomogeneity of the skull structure and the heterogeneity of the material, ultrasonic wave transmissions through the skull are tremendously attenuated and distorted [8], which requires effective numerical model to simulate the ultrasonic field characteristics of transcranial ultrasonic focusing.

Time-reversal methods and phased-array methods have been primarily used in previous transcranial ultrasound focusing researches. The time reversal technique can realize good focusing effects in transcranial ultrasound [9,10], but its complicated emission waveform brings inconvenience to practical application. Ultrasound phased array technology with a simple emission waveform and flexible beam control is very popular in practical applications. There are large numbers of experimental and theoretical studies on transcranial ultrasound phased array focusing. Hynynen et al. [11,12,13] proposed the implanted hydrophone method in which a hydrophone was placed at the focal point in an isolated skull. The hydrophone was used to measure, invert, and applied the phase shift induced by the presence of the skull to all the elements of the array. A 64-element 0.664 MHz hemispherical array was used to focus at a distance of 10 mm on the other side of the skull to coagulate tissue. Further, Aubry [14] proposed a method for calculating the acoustic properties of the skull from CT scans. The numerical simulation model of the skull based on CT data allowed the calculation of the transducer-to-focus time delay used for the experiment. Based on the modeling of the CT data, Clement and Hynynen [15] introduced projection algorithms to calculate the excitation phase of each transducer element. The calculated excitation phase was applied to 0.74 MHz, 320-element hemispherical array emitted through the human skull with the focus shift within 1 mm. Marquet [16,17] performed noninvasive ultrasound tissue ablation based on time delays calculated from numerical models. In vitro experiments on monkey and human skull samples using an array of 300 emitters centered at 1 MHz revealed localization errors of less than 0.7 mm. Also, numerical modeling methods for sound field computation were well discussed and compared. Jing [18] compared the k-space method with the finite-difference time-domain (FDTD) method. The results showed that the two methods match well in model calculations with more than 10 grid points per wavelength. However, when the grid spacing was increased, the k-space method produces much smaller numerical errors. Jiang [19] compared the phase calculation results of the k-space, FDTD, and ray-tracing methods in transcranial ultrasound phase array focusing. He found that the phase errors of the k-space, FDTD, and ray-tracing methods were 0.7%, 1.2%, and 5.35%, respectively. This shows that the k-space method is a very effective calculation method in transcranial ultrasonic simulation.

The structure of the skull is relatively complex, including the inner and outer layers of cortical bone and the middle layer of trabecular bone. The cortical bone is dense bone tissue characterized by low porosity, whereas trabecular bone is highly porous and fluid filled. The numerical model of transcranial ultrasonic focusing can be obtained by CT images [20]. However, the general clinical CT images have a lower resolution (is usually 0.488 mm–0.625 mm) [21]. The average size of trabecular bone ranges from 50 µm to 150 µm, and the spacing of the bone layers range from 0.5 mm to 2 mm [22]. The pores and bone layer structures in trabecular bone are missing and trabecular bone is approximately a low-velocity homogeneous medium layer. Therefore, it is difficult to accurately estimate ultrasound propagation and attenuation characteristics in transcranial ultrasound models constructed by the clinical CT images.

In order to display the structure of pores and bone layers in trabecular bone, Bossy [23] used micro-computed tomography (micro-CT) to numerically model trabecular bone. His research results indicated that the microstructures of trabecular bone caused multiple scattering, which greatly attenuated ultrasonic waves. Pinton [24] verified this conclusion with experiments and showed that only a small portion of the loss in transcranial ultrasonic focusing was caused by bone absorption. Robertson [25] revealed that ignoring the microstructures of trabecular bone in transcranial skull modeling led to errors in the calculation of attenuation and propagation time of ultrasonic wave. Robertson’s research showed that the relative error between the ultrasound amplitude calculated by the clinical CT model for the transmitted skull was over 60% from the actual amplitude, and the time-of-flight error for the transmitted skull was over 0.3 µs. In summary, transcranial ultrasound focusing is able to achieve a focusing error of approximately 1 mm based on a numerical model established by clinical CT. However, the clinical CT model calculations ignore the scattering from bone trabecular structures and cannot accurately estimate the range of the focusing focal spot. Therefore, in order to accurately calculate the loss and propagation of ultrasound within the skull, transcranial ultrasound modeling should take into account the effect of trabecular structures in model calculations. However, there are few studies and discussions on transcranial ultrasound focusing for micro-CT modeling.

In this paper, we use micro-CT images to reconstruct micro-CT models with trabecular bone pores and bone layer structures. In order to distinguish between bone trabecular structures with consideration of issues such as computational efficiency, we chose the resolution for micro-CT as 60 μm, which is sufficient for micro-CT images to show the intracranial foramina and bone layer structure. Based on the micro-CT model, we investigate the effect of bone trabecular microstructure on ultrasound propagation characteristics. Since the microstructure of the skull causes distortion of the ultrasound waveform as it transmits through the skull, we apply the pulse compression to the calculation of micro-CT models, which has been used in medical ultrasound to improve the accuracy of delay calculations [26]. The pulse compression method calculates the time delay from the transducer array to the focusing point with high computational accuracy. The delay setting calculated by the pulse compression method makes phased array focusing possible in the micro-CT model. The effects of signal frequency, depth of focus, beam deflection and amplitude adjustment on the phased array focusing effect are investigated.

## 2. Modeling Methods and Calculation Results

### 2.1. Establishment of the Transcranial Model

**Figure 1:**
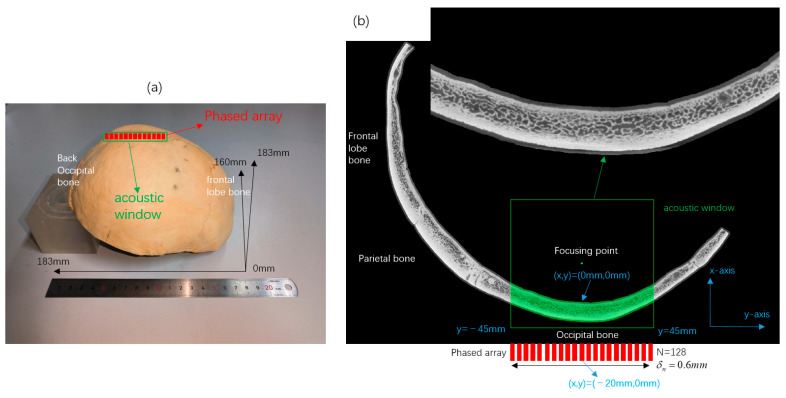
(**a**) Schematic representation of the skull scanning region, the acoustic window and the position of the phased array. (**b**) Skull slice images, acoustic window and line array coordinate positions.

**Figure 2:**
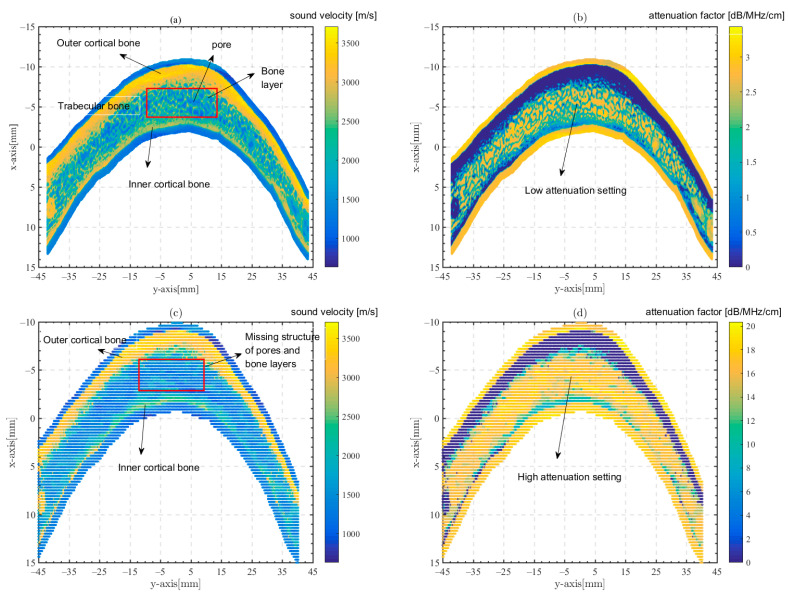
Bone structure and acoustic parameters in models. (**a**,**b**) The micro-CT model, (**c**,**d**) the clinical CT model, (**a**,**c**) sound velocity c and (**b**,**d**) the attenuation factor α.

The micro-CT model is based on the CT scan of an ex vivo skull. The scanning equipment is YXLON FF85, a high-power, high-precision computed tomography system. The tube voltage of the scan is 210 kV, and the tube current is 112.0 uA. The X-rays used for the scan are cone beams, and the scan is rotated by the circle trajectory method. The reconstruction algorithm of the CT images is Feldkamp. Figure 1a shows a schematic of the skull, the skull is swept with the occipital bone padded 50 mm high. The anterior–posterior range of the sweep is 183 mm, the left–right range is 183 mm, and the vertical height is 160 mm, with a resolution of 60 µm in each direction. We take one slice in the middle of the skull, as shown in Figure 2b. The acoustic window is set at the occipital bone of the slice. We establish a Cartesian coordinate system (x, y) using the junction of the inner skull and brain as the coordinate origin. The positive x-axis is toward the deep brain. The transducer array with 128 elements is placed along the y-axis, and the coordinate of the transducer center is (−20 mm, 0 mm). The range of the transducer array is (−20 mm, −38.4 mm~38.4 mm) and the spacing of each element is 0.6 mm. The simulation environment is a skull placed in pure water, as shown in Figure 1, where the pores of the skull are filled with aqueous medium.

It can be seen that the skull is composed of inner and outer cortical bone and middle trabecular bone, with complex bone layers and various sizes of pores forming the trabecular bone microstructures.

The micro-CT image provides the distribution of Hounsfield values. The acoustic parameters of the skull can be obtained by the Hounsfield value. At first, the porosity ϕ of the skull is calculated by the Hounsfield value H of each pixel point in the CT image by:

(1) $$\phi =1-\frac{H}{1000}$$

Then, the density ρ, sound velocity c and the attenuation factor α at each point of the grid in the skull model are calculated as follows [14,19,27,28]:

(2) $$\left\{\begin{array}{l} \rho =\phi \rho_{w}+(1-\phi )\rho_{b},\;\\ c=c_{w}+(1-\phi )(c_{b}-c_{w}),\;\\ \alpha =\phi^{0.5}\alpha_{b}.\; \end{array}\right.$$

where $\rho_{w}$ and $c_{w}$ are the density and sound velocity of the water, $\rho_{b}$ and $c_{b}$ are the density and sound velocity of the skull, respectively, and $\alpha_{b}$ is the attenuation factor of the skull. In the calculation, $\rho_{w}=1000{\;\mathrm{kg}/m}^{3}$, $\rho_{b}=1900{\;\mathrm{kg}/m}^{3}$, $c_{w}=1500\;m/s$, and $c_{b}=2900\;m/s$. The attenuation $\alpha_{b}$ in Equation (2) is the maximum absorption attenuation value in the skull. In the micro-CT model, the skull is considered as porous media, including the bone medium and water in the pores. Therefore, the largest attenuating medium is bone medium, with an $\alpha_{b}$ value of 2.7 dB/cm/MHz as measured in the literature [24] for cortical bone resorption.

The acoustic parameters of the skull can be calculated by Equation (2). Figure 2 gives the bone structure and ultrasonic parameters obtained by Equation (2). In order to investigate the effect of missing trabecular bone microstructures in the transcranial model, we eliminate the porosity and bone layer structure of the trabecular bone by reducing the resolution and homogenizing the micro-CT model, and establish a model similar to the clinical CT image. The model is a clinical CT model. The bone attenuation coefficient $\alpha_{b}$ for the clinical CT model is 16 dB/cm/MHz [19], and the sound velocity and density are calculated in the same way as for the micro-CT model. The bone structure and acoustic parameters of the clinical CT model are also shown in Figure 2.

It can be seen that the skull has a complex structure but the attenuation factor of the bone is low in the micro-CT model as shown in Figure 2a,b. The skull is missing the trabecular bone structure while the attenuation factor of the bone is set higher in the clinical CT model as shown in Figure 2c,d.

### 2.2. Calculation of the Ultrasonic Field

The pseudospectrum approach [29,30], the finite difference method [31], the angular spectrum method [32], and other methods of computation can be used to calculate the transcranial ultrasound model. Aubry [33] compared various modeling and calculation methods, and the result showed that the above calculation methods have good consistency. The calculation method based on the k-wave toolbox was used as the benchmark for validation. The pseudospectral approach and first-order fluid coupling equation are the bases for the model computation in this article, which also uses the k-wave toolbox. The calculation equation is as follows [30,34]:

(3) $$\left\{\begin{array}{l} \frac{\partial v}{\partial t}=-\frac{1}{\rho_{0}}\nabla p,\\ \frac{\partial \rho }{\partial t}=-\rho_{0}\nabla \cdot v-v\cdot \nabla \rho_{0},\\ p=c_{0}{}^{2}(\rho +u\cdot \nabla \rho_{0}-\tau \frac{\partial }{\partial t}{\left(-\nabla^{2}\right)}^{\frac{y}{2}-1}\rho -\eta {\left(-\nabla^{2}\right)}^{\frac{y+1}{2}-1}\rho ). \end{array}\right.$$

where v is the velocity of the particle, $\rho_{0}$ is the static density, ρ is the density of the medium, $c_{0}$ is the isentropic sound speed, and u is the displacement vector of the particle. τ and η are the absorption and dispersion scaling factors. where $\tau =-2\alpha c_{0}^{y-1},\eta =2\alpha c_{0}^{y}\tan(\pi y/2)$. These two items in the calculation control the frequency dependence of the ultrasonic attenuation and the dispersion of the waveform, respectively. Since the dispersion of sound waves is not considered in transcranial ultrasound simulation [35]. Therefore, y is set to a value close to 1 in the calculation to eliminate waveform dispersion in the calculation, which is taken here as y=1.05 [36].

In the calculation, the time step is reduced to ensure the convergence of the calculation results, and the time precision is set based on the Collan–Friedrich–Levy condition number (CFL). The time step Δt is calculated by the maximum speed of sound in the medium $c_{\max}$, the spatial grid resolution Δr and the Courant–Friedrichs–Lewy condition number $N_{CFL}$. The grid resolution in the micro-CT model is 60 μm, while the grid resolution in the clinical CT model is 0.3 mm. Both computational models satisfy the requirement of 3 grid points per wavelength for the k-wave method. To ensure that the calculation results converge accurately, we determine the convergence time step values for the two model calculations by iterative time steps. The convergence value of the time steps is Δt=2.5 ns in the micro-CT model and Δt=8 ns in the clinical CT model, respectively. The actual $N_{CFL}$ of the corresponding models is $N_{CFL}=0.15$ and $N_{CFL}=0.1$, respectively. The time steps are calculated as follows [37]:

(4) $$\Delta t=\frac{N_{CFL}\Delta r}{c_{\max}}$$

Shear waves are not considered in the calculation of the model in this paper. In previous studies, the skull was considered to be an isotropic solid medium [38,39,40,41]. But in fact, the excitation of the shear wave requires a large incidence angle. Shear waves require an incidence angle greater than 35° for effective excitation, and the excitation amplitude is greater than that of longitudinal waves at incidence angles greater than 40° [42,43]. In the 128-element line array used in the computational model of this paper, the incident angle of the farthest edge array unit is 35°. Therefore, during the focusing process, the acoustic waves in the skull are dominated by longitudinal waves. The attenuation of the shear wave in the skull is much higher than that of the compression wave, so the shear wave is usually not used as a focusing wave considering shear waves in the skull model can accurately estimate the energy of the focal point [44].

In the micro-CT model, the pores in the bone structure are saturated fluid pores with greater shear wave propagation attenuation as well as more complex waveform conversion. It is assumed in this study that the shear wave is greatly dissipated during the transmission of the cranial bone, and does not affect the focusing quality of the focal point. Meanwhile, the nonlinear setting in the transcranial model calculation does not affect the calculation of the time delay and the time-reversal focusing effect [45], so the nonlinear term is ignored in the calculation.

### 2.3. Influence of Microstructure of Trabecular Bone on Ultrasonic Loss Calculations

In order to calculate the ultrasonic loss in the skull, we excite ultrasonic signals through the transducer array shown in Figure 1. We use one-dimensional line arrays because they are easy to fabricate and achieve good focus variability. The line arrays are located above the skull and each array element is excited sequentially. High-frequency signals used in transcranial ultrasound focusing with higher attenuation but better focusing. Higher-frequency transducer arrays have a smaller size and better flexibility. Therefore, compared to the 500 KHz frequency used for clinical transcranial focusing, we choose 1 MHz frequency by considering the focusing effect, focusing efficiency and array size. The excitation signals are four-period cosine envelope (CE) signals with a frequency of 1 MHz. The skull and transducer array are placed in the water, and the received signal at the focusing point is calculated from Equation (3).

Based on the above settings, the maximum amplitude of the received signal at the focusing point is $A_{b}$, while the maximum amplitude of the received signal at the focusing point in the absence of the skull existence is $A_{w}$. The ultrasonic loss in the skull is $\alpha_{l}=20\mathrm{lg}(A_{b}/A_{w})$. The ultrasonic loss from each array element to the focusing point is calculated by the above method.

**Figure 3:**
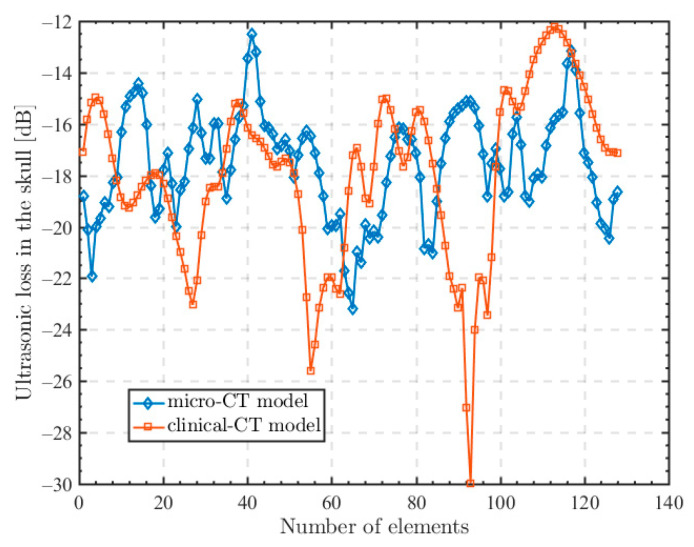
Ultrasonic loss from each array element to the focusing point.

The ultrasonic losses from each array element of the transducer to the focusing point are calculated in the micro-CT model and the clinical CT model using the above calculation process and the result is shown in Figure 3. The ultrasonic losses in the micro-CT model are −(16~20) dB and in the clinical CT model are −(12~28) dB. It can be seen that the ultrasonic losses in the micro-CT model come from the scattering of the trabecular bone pore and the bone layer. In contrast, due to the lack of the microstructure of the trabecular bone, the clinical CT model is set by a higher attenuation value to fit the attenuation due to scattering. 

Laurent [46] measured the ultrasonic attenuation in six isolated skulls in the frequency range of 800 kHz–1.3 MHz. The results show that the ultrasonic attenuation of 1 MHz frequency signal transmitting through the skull is −(10~16) dB. The average ultrasonic attenuation of a 1 MHz frequency signal through the skull computed by the micro-CT model is −16 dB with a variance of 2.0. However, if the clinical CT model is used for the calculation, the attenuation of the 1 MHz frequency is −17 dB with a variance of 4.3, which is more than twice that of the micro-CT model.

### 2.4. Characterization of the Transmitted Skull Ultrasound Signal

**Figure 4:**
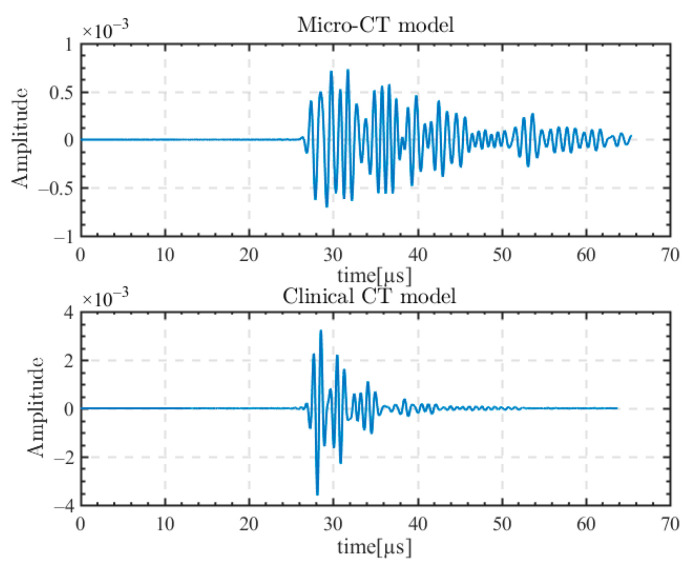
Transmission skull ultrasonic waveforms received at the focusing point for the micro-CT model and the clinical CT model.

Due to the presence of complex trabecular bone structures in the micro-CT model, the ultrasound waveforms transmitting through the skull produce distortions and multiple scattering. We excited 1 MHz CE signal on the 64th array element in the center of the array, and the excited ultrasound signal indicated a near-vertical incidence. Figure 4 shows the ultrasonic waveforms received at the focusing point for the micro-CT model and the clinical CT model.

The signal received at the focusing point in micro-CT is the superposition of waveforms after multiple scattering. The superposition of scattered waves not only causes distortion of the received waveform, but also increases the time domain length of the signal. In the clinical CT model, the received ultrasonic waveform at the focal point is more complete, without strong distortion and obvious trailing.

**Figure 5:**
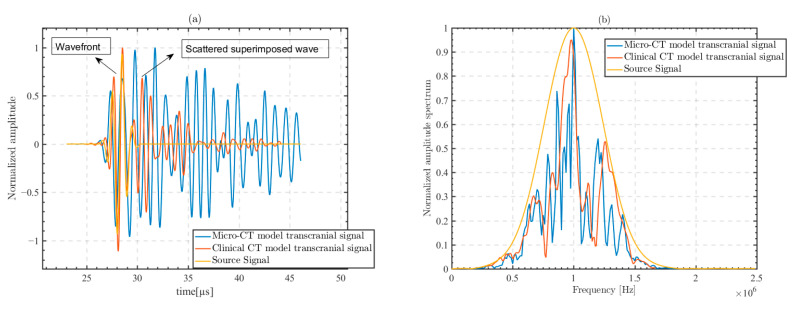
Comparison between the transcranial skull signal received at the focusing point and the excitation source signal of the transducer in the micro-CT model and the clinical CT model. (**a**) Signal waveforms and (**b**) signal spectra.

Figure 5 shows the characteristics of the ultrasonic signal at the focusing point in the micro-CT model and clinical CT model. Figure 5a shows the waveform comparison between the transmitted skull ultrasonic signal and the excitation signal. Figure 5b shows the spectral comparison of the transmitted skull ultrasonic signal and the excitation signal.

The distortion of the waveform is caused by several factors. The bone structure leads to an increase in the nonuniformity of the propagation medium. The increase in the resolution of the modeled computational grid and the increase in the differences in acoustic parameters between grid points lead to distortions in the shape of the waveform. The bone layer in the trabecular bone scatters the transcranial ultrasonic waves many times [22,47]. The received signal at the focusing point becomes the superposition of multiple scattered waves. Therefore, the transcranial waveforms in the micro-CT model are more complex than those in the clinical CT model, and have larger aberrations and scattering.

Furthermore, the computational model takes into account the positive frequency dependent attenuation specific to biological tissues. That is, the attenuation coefficient has a high attenuation value in the high-frequency part of the signal [23,48], which leads to a frequency shift in the center of the signal. There are spectral aberrations in the transcranial signals of both the micro-CT model and the clinical CT model.

## 3. Application of Pulse Compression in Transcranial Phased Array Focusing

Ultrasonic phased array focusing controls the time delay of the ultrasonic signal of the array element so that the ultrasonic signal reaches the focusing point at the same time [49]. Ultrasonic phased array can focus ultrasonic waves in various complex media [50]. For uniform media, the time delay of each array element of the transducer can be obtained from the distance and velocity. For nonuniform media, ultrasonic phased array can calculate the time delay from the transducer to the focusing point by the cross-correlation of the excitation signal of the transducer and the received signal at the focusing point.

However, it is very difficult to accurately calculate the time delay from the transducer to the focusing point in the transmitted skull ultrasonic waveform shown in Figure 5a. Since the ultrasonic shape is distorted and scattered, it is difficult to obtain an accurate time delay by the cross-correlation based time delay method. Therefore, we employ the pulse compression method to improve the accuracy of this time delay calculation. The pulse compression method changes the autocorrelation function of the original signal. The autocorrelation function of the conventional unmodulated CE signal is still the envelope shape, and the peak of the cross-correlation is the maximum of the envelope. Pulse compression changes the autocorrelation function of the signal to peak pulse. The autocorrelation function of the spikes is effective in resisting frequency shifts, while the phase encoding in the pulse compression method eliminates the effect of the scattered wave cross-correlation peaks. Moreover, the large time-width and bandwidth signal modulated by the pulse compression method can obtain the signal-to-noise ratio of the matched filter and improve the robustness of the cross-correlation.

### 3.1. The Principle of Pulse Compression

The pulse compression method modulates the excitation ultrasound signal into a long-duration waveform by frequency modulation and phase coding, and decouples the received ultrasound signal into a narrow-band pulse with a high signal-to-noise ratio by using cross-correlation at the focusing point [51,52,53,54].

The pulse compression method constitutes the matching filter of the excitation ultrasonic signal and the received signal at the focusing point. The output gain of the matching filter is equal to the time bandwidth product of the excitation ultrasonic signal. Therefore, the pulse compression method actually increases the output gain of the matched filter by increasing the time bandwidth product of the excitation ultrasonic signal and thus further increases the accuracy of the time delay calculation.

The time bandwidth product of a monofrequency ultrasonic signal is a fixed value due to the fact that the signal is periodically broadened in the time domain and its bandwidth in the frequency domain decreases [55]. The time bandwidth product of an ultrasonic signal can only be increased by frequency modulation and phase coding. Frequency modulation and phase coding can increase the root-mean-square signal bandwidth β and the root-mean-square signal duration δ of the signal, respectively, consequently increasing the time bandwidth product $T_{B}$ of the excitation signal p(t) with the following expressions [56]:

(5) $$\left\{\begin{array}{l} \delta^{2}={\left(\frac{2\pi }{\sqrt{2E}}\right)}^{2}\int_{-\infty }^{\infty }t^{2}|p(t){|}^{2}dt\\ \beta^{2}={\left(\frac{2\pi }{\sqrt{2E}}\right)}^{2}\int_{-\infty }^{\infty }f^{2}|P(f){|}^{2}df\\ T_{B}=\delta^{2}\cdot \beta^{2} \end{array}\right.$$

In the above equations, P(f) is the Fourier transform of p(t) and E is the energy of the signal p(t). For a matching filtering process based on the cross-correlation calculation, the signal-to-noise ratio gain $G_{SNR}$ is expressed as [55]:

(6) $$G_{SNR}=\frac{(S\cdot T)/N_{0}}{S/(B\cdot N_{0})}=T\cdot B=T_{B}$$

In the matched filter expression of the above equation, $N_{0}$ is the noise power spectrum density, S is the average power of the excitation signal, T is the total duration of the excitation signal, and B is the bandwidth of the excitation signal. The time bandwidth product $T_{B}$ of the excitation signal is often used in the literature to describe the $G_{SNR}$ of the matched filtered output.

### 3.2. Design of the Linear Frequency Modulation Barker (LFMB) Code

The linear frequency modulation (FM) signal bandwidth is insensitive to the target doppler frequency deviation, but it has the disadvantage of strong time-delayed frequency-deviation coupling, which cannot resolve the effect of the presence of scattered waves in the signal. The Barker code is a nonperiodic sequence with good autocorrelation properties, which can reduce the computational error caused by scattered waves. Therefore, the phase modulation of the signal in the Barker code, followed by modulation of each code element on linear FM, can be computationally robust to the superposition and frequency shift of scattered waves in the transcranial signal.

**Figure 6:**
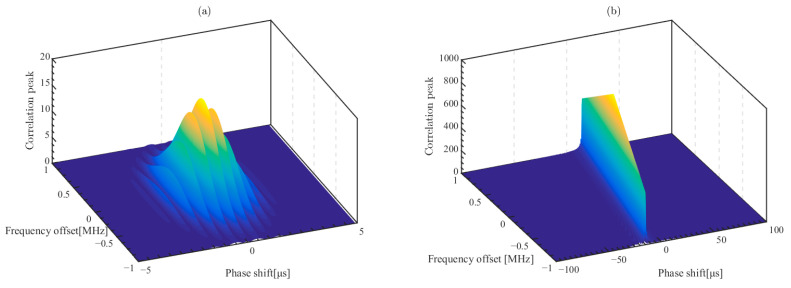
The ambiguity function of the excitation signal. (**a**) The unmodulated CE signal; (**b**) the frequency-modulated signal.

Figure 6 shows the unmodulated CE signal and the frequency-modulated signal with the ambiguity function. The ambiguity function of the excitation signal represents the output response of this signal in the matched filtering with the expression:

(7) $$\chi (\tau ,f_{d})=\int_{-\infty }^{\infty }p(t)\cdot p(t–\tau )\cdot e^{2\pi if_{d}t}dt$$

Here, $\chi (\tau ,f_{d})$ is the ambiguity function of the excitation signal p(t), τ is the time delay and $f_{d}$ is the frequency offset. The ambiguity function of the unmodulated CE signal in Figure 6a shows that the maximum output value is obtained at $f_{d}=0,\tau =0$. However, when the received signal with time delay τ includes the scattered signal with frequency offset $f_{m}$ and phase shift $\tau_{m}$, the output of matched filtering is $\chi (\tau +\tau_{m},f_{m})$, resulting in $\chi (\tau +\tau_{m},f_{m})>\chi (0,0)$. The time delay obtained by the cross-correlation calculation is erroneous.

Figure 6b shows the ambiguity function of the excitation signal after frequency modulation. The gain of the signal time bandwidth product is expressed as the increase in the ambiguity function at χ(0,0). Frequency modulation also changes the shape of the ambiguity function. The ambiguity function of the frequency-modulated signal is the the coupling of phase shift and frequency offset, thus the frequency modulation reduces the computational error caused by the frequency offset. The ambiguity function with coupling coefficient κ can be expressed as the following equation.

(8) $$\chi (\tau ,f_{d})=\left\{\begin{array}{l} \chi (\tau ,f_{d}),\tau =\kappa f_{d}\\ 0,\tau \neq \kappa f_{d} \end{array}\right.$$

Since the transcranial ultrasonic signal is a superposition of waveforms that have been scattered many times, and the frequency-modulated signal will be similarly distorted due to scattering, we use the frequency-modulated ultrasound signal as a two-phase encoded codeword in order to achieve phase coding. Phase coding further reduces the narrow pulse width of the frequency-modulated signal at the matched filter output.

The 13 bit Barker code is used as a binary code set for phase encoding. The frequency-modulated signal with 8 µs length, 1 MHz center frequency, and 2 MHz bandwidth is used as a single code element to constitute the linear frequency modulation Barker (LFMB) code. The total length of the LFMB code is 104 µs, and the LFMB code is used in simulation to improve the accuracy of time delay calculations, which is expressed as [57]

(9) $$\left\{\begin{array}{l} p_{1}(t)=\sin(2\pi (f_{c}-\frac{B}{2})t+\pi \mu t^{2}),t\subset [0,t_{L}]\\ p_{2}(t)=\sum_{m=0}^{L-1}C_{m}\delta (t-mt_{B})\\ C_{m=0-12}=\left[+1+1+1+1+1-1-1+1+1-1+1-1+1\right]\\ p(t)=p_{1}(t)⊗p_{2}(t) \end{array}\right.$$

The LFMB code is computed by convolving the linear frequency modulation signal $p_{1}(t)$ with the Barker code $p_{2}(t)$. ⊗ is the convolution symbol, $f_{c}$ is the center frequency, B is the bandwidth, $t_{L}$ is the length of linear frequency modulation signal, $\mu =\frac{B}{t_{L}}$ is the frequency modulation slope, and $t_{B}$ is the length of the LFMB code. $C_{m}$ is the symbol of the 13 bit Barker code elements. 

**Figure 7:**
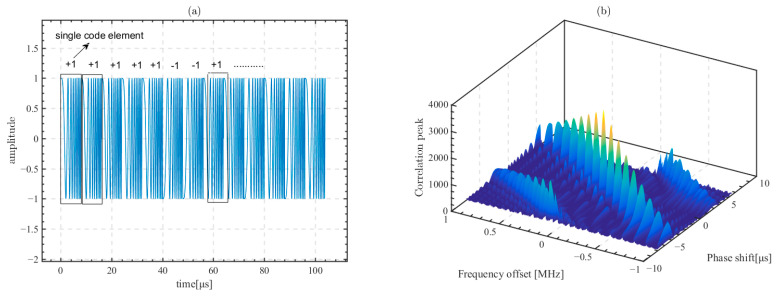
Signal characteristics of the LFMB code. (**a**) The time domain signal; (**b**) the ambiguity function.

Figure 7a shows the time domain waveform of the LFMB code and Figure 7b shows the ambiguity function of the LFMB code. It can be seen that phase coding discretizes the coupling of the frequency offset and the phase shift, which reduces the computational error and further improves the time bandwidth product at the same time.

### 3.3. Transcranial Ultrasound Phased Array Focusing Based on the Pulse Compression Method

In the simulation micro-CT model, we transmit the excitation ultrasonic signal p(t) at each array element of the transducer. The excitation signal p(t) is the LFMB code calculated from Equation (9). For convenience, the excitation signal of the jth element is represented by $p_{j}(t)$. Under the excitation of this signal, the ultrasonic field generated at the focal point is represented by $p_{o}(t)$ which can be calculated according to Equation (3). The waveform of $p_{o}(t)$ is distorted when the ultrasonic wave is transmissive through the skull. However, the signal $p_{o}(t)$ can be decoupled into a single pulse by calculating the cross-correlation of $p_{j}(t)$ and $p_{o}(t)$, as shown in the following equation

(10) $$R_{j}(\tau_{j})=\int_{-\infty }^{\infty }p_{j}(t)p_{o}(t+\tau_{j})dt$$

$R_{j}(\tau_{j})$ is the cross-correlation function of the jth element, and the time delay $\tau_{j}$ from the jth array element to the focusing point can be obtained from the peak value of the decoupled pulse. In actual transcranial ultrasound phased array focusing, the transducer array is based on the time delay settings calculated in the above model to make the emitted focusing ultrasonic waves reach the focusing point. The excitation signal $p_{j}(t)$ in the model calculations is a long-duration modulated signal and is difficult to emit with the actual transducer. Therefore, in phased array focusing, the excitation signal of the jth array element adopts a single-frequency signal $f_{j}(t)$ with two cycles.

**Figure 8:**
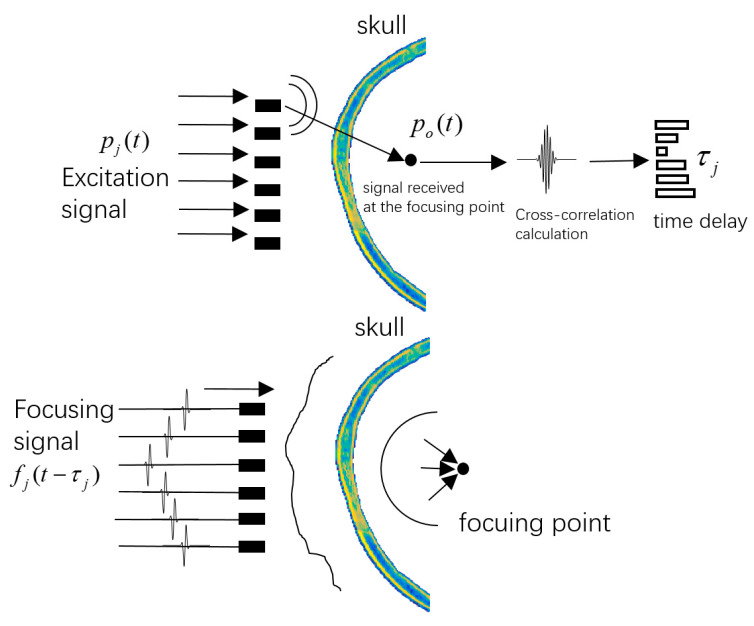
Transcranial ultrasound phased array focusing based on the pulse compression method.

We perform phased array focusing based on the time delay calculated by Equation (10). Thus, each element of the transducer emits signal $f_{j}(t)$ and the time delay is given by Formula (10). Therefore, the excitation signal of the jth array element is $f_{j}(t-\tau_{j})$. When all array elements simultaneously emit this signal $f_{j}(t-\tau_{j})$, the ultrasonic field is focused at the focal point, as shown in Figure 8.

## 4. Simulation Results

### 4.1. Comparison of Time Delay Calculations

**Figure 9:**
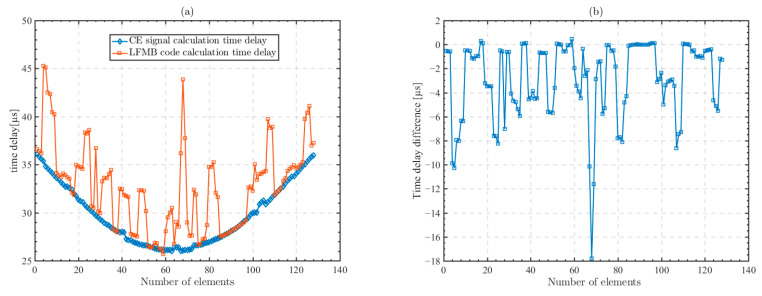
Time delay of each array element computed by the unmodulated CE signal and the LFMB code and the difference between the computed time delays of the two signals. (**a**) Time delay of each array element; (**b**) difference between the computed time delays of the two signals.

Following the method shown in Figure 8, in the micro-CT model, we excite the unmodulated CE signal and the LFMB code on each array element of the transducer. The time delay from each array element to the focusing point is calculated according to Equation (10). Figure 9a shows the time delays calculated by the unmodulated CE signal and the LFMB code, respectively. Some of the time delays calculated by the two excitation signals are equal, while most of the time delays show a large deviation in the calculated values, and the deviation values ranging from 0 to 10 μs.

Figure 9b shows the difference between the time delays calculated for the two excitation signals. When the received waveforms are not distorted due to the frequency offset and the superposition of the scattered waveforms, the calculated time delays of the two excitation signals are the same. Where the cross-correlation calculation produces an error due to frequency offset, the difference obtained from the calculation is between 0.5 and 1 μs; and when the received waveform is peak lagged due to distortion and scattered wave superposition, the calculation difference is greater than 1 μs.

**Figure 10:**
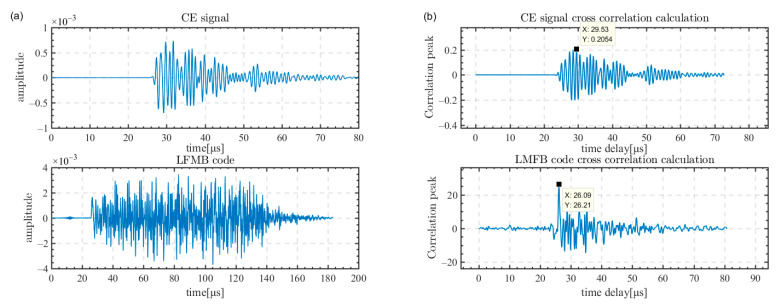
Received signal waveforms of the excitation signal at the focusing point and the results of the cross-correlation calculation of the received signals. (**a**) Received signal waveforms at the focusing point of the unmodulated CE signal and the LFMB code; (**b**) results of the cross-correlation calculation of the received signals of the unmodulated CE signal and the LFMB code.

In order to show more visually the computational error of transcranial ultrasound distortion visually, Figure 10a shows the received waveforms of the unmodulated CE signal and the LFMB code excited at the same array element as the focusing point. The position of the 64th array element is in the center of the array, and the excitation signal is close to the vertical incidence on the outer surface of the skull. It can be seen that the two excitation signals are distorted due to the scattering after transmission through the skull in Figure 10a. The cross-correlation calculation results of the two signals are shown in Figure 10b. The time delays are 29.53 μs for CE signal and 26.09 μs for the LFMB code, and the calculation difference is 3.44 μs.

In Figure 10b, the correlation peak of the CE signal is 0.2045, while the correlation peak of the LFMB code is 26.21, which indicates that the increased time bandwidth product of frequency modulation and phase coding increases the correlation peak of the correlation computation. We measure the accuracy of the time delay calculation by the peak sidelobe ratio of the cross-correlation calculation. The peak sidelobe ratio is the ratio of the highest peak to the second highest value in the cross-correlation calculation. The higher the peak sidelobe ratio, the higher the calculation accuracy.

The second highest correlation peak of the CE signal is 0.2004, and that of the LFMB code is 13.53. The peak sidelobe ratio of the CE signal is 20 × log10(0.2054/0.2004) = 0.21 dB, and the peak sidelobe ratio of the LFMB code is 20 × log10(26.21/13.53) = 5.74 dB. Pulse compression coding increases the peak sidelobe ratio to 5.53 dB.

### 4.2. Phased Array Focusing with Different Time Delays

**Figure 11:**
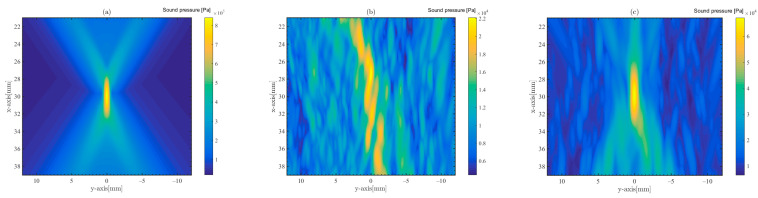
Maximum sound pressure sound field plot for phased array focusing based on different time delay settings, with the origin of the plot located 50 mm below the center array element of the transducer array. The frequency of the focusing ultrasonic waves is 1 MHz. (**a**) Phased array focusing in the absence of the skull environment in water, (**b**) focusing on time delay calculated from the unmodulated CE signal, and (**c**) focusing on time delay calculated from the LFMB code.

With the time delay settings in Figure 9a for phased array focusing, the signal used for focusing is a 1 MHz pulse wave. The sound pressure of the pulse wave excited on each array element is 1 MPa, and the depth of focus is 30 mm below the skull. Figure 11 shows the maximum sound pressure value map at each point within the sound field of the focusing point. The x-axis direction in the figure is the depth direction, and the greater positive direction of the x-axis indicates that the focusing point is farther away from the skull surface and deeper. The y-axis direction is the transverse direction, and the line array is symmetric about the y-axis. The origin in Figure 11 is located at a position 50 mm below the array center array element, i.e., 30 mm below the skull.

Figure 11a represents phased array focusing in the absence of the skull environment in water. Figure 11b,c are focusing distribution in the skull in water. Figure 11b shows the distribution of the phased array focusing sound field with the time delay obtained from unmodulated CE signal acquisition, and no effective focus. Figure 11c shows the phased array focusing sound field distribution with time delay obtained from the LFMB code. When the time delay is calculated accurately, the phased-array method can achieve effective focus in the micro-CT model.

This shows that phased array focusing can still be realized in the micro-CT model of complex skull structures with precise delay settings. Taking the −3 dB width of the peak sound pressure at the focal point as the effective range of the focused beam, it can be found that the −3 dB widths in the x-axis and y-axis directions at the focal points in Figure 11a are 4.0 mm and 0.8 mm, respectively, while in Figure 11c, the −3 dB widths in the x-axis and y-axis directions at the focal points are 6.3 mm and 1 mm, respectively. Therefore, the beam widths transmitted through the skull are broadened.

### 4.3. Effect of Signal Frequency on Focusing

**Figure 12:**
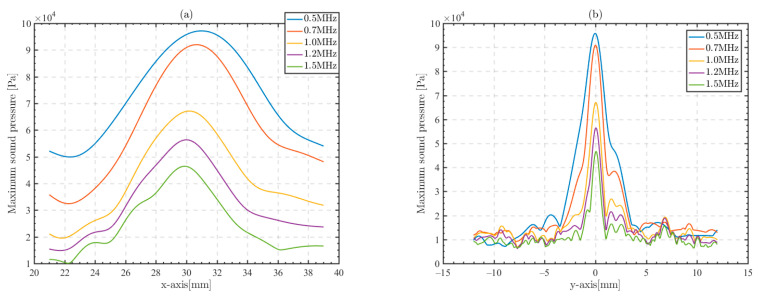
Focused beams of different frequency focusing signals at the time delay setting of the LFMB code calculation. (**a**) X-axis beamwidth; (**b**) y-axis beamwidth.

Figure 12 shows the variation in the focused beam at different frequencies, and the beam width decreases significantly in both the y-axis and the x-axis as the frequency increases, while the sound pressure at the focusing point decreases with increasing frequency. Among them, there is significant attenuation at 1 MHz.

In the preceding content analysis, the cranial medium of the micro-CT model has low attenuation values and an increase in frequency does not significantly increase attenuation absorption. Therefore, the decrease in sound pressure values is due to the enhanced scattering of the signal in the trabecular bone as the wavelength is reduced [10]. When the focusing frequency is increased from 0.5 MHz to 1 MHz, the sound pressure of the focal point decreases by −3.2 dB, the x-axis −3 dB beamwidth decreases from 9.7 mm to 6.3 mm, and the y-axis −3 dB beamwidth decreases from 2.3 mm to 1 mm, whereas when the focusing frequency is increased to 1.5 MHz, the x-axis −3 dB beamwidth is 4.4 mm, the y-axis −3 dB beamwidth is 0.6 mm, and the focal sound pressure decreases by −6.4 dB compared to that at 0.5 MHz.

### 4.4. Focus Depth Modulation and Beam Deflection

**Figure 13:**
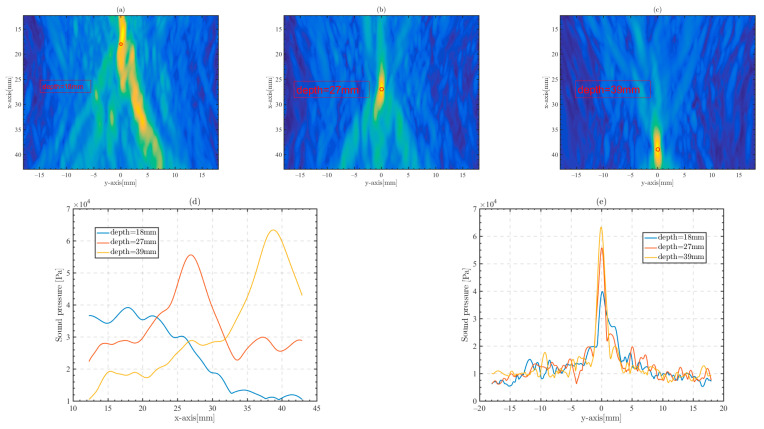
Focusing at different depth positions (red circles) by the phased-array method. (**a**–**c**) The two-dimensional focusing ultrasonic field distribution with a focus depth of 18 mm, 27 mm, and 39 mm, respectively. (**d**,**e**) The x-axis and y-axis ultrasonic beamwidths with different depth positions.

Ultrasound phased array technology can adjust the depth of the focusing point by setting time delays or adjusting the position of the focusing point by using beam deflection. The time delays for the different focusing points are similarly calculated from the LFMB code. Figure 13 shows the focusing effect at different depths under the skull through phased array in the micro-CT model. The depth of the focusing point is 18 mm, 27 mm and 39 mm, respectively. Figure 13a–c shows the focused beams of the phased array at different depths, and the red circle marks the location of the focusing point.

Figure 13d,e show the x-axis and y-axis beamwidths of focal points at different depths, respectively. Focusing at a shallow depth of 18 mm resulted in a focus shift, which is due to the larger incidence angle of transducer array elements at shallow focal points. At the same time, with the increase in focus depth, the beam width of the focus widened, and the peak sound pressure of the focus increased. This suggests that the ultrasonic phased array is more suitable for focusing in the deep brain, rather than the shallow cerebral cortex.

**Figure 14:**
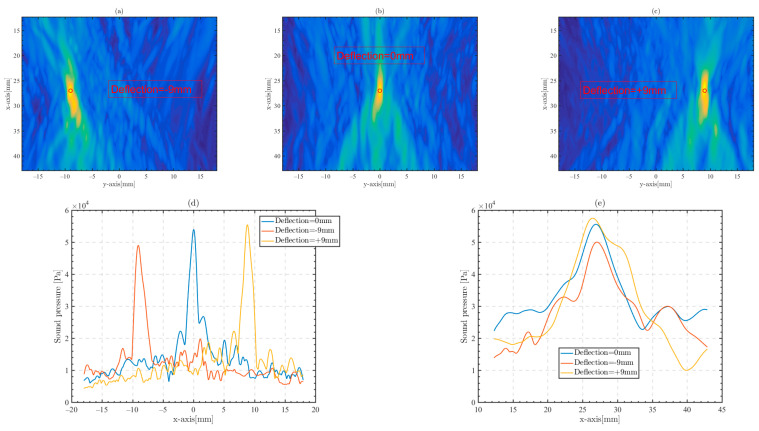
Beam deflection to achieve phased array focus at different focus positions (red circle). (**a**) Deflection = −9 mm, (**b**) deflection = 0 mm, (**c**) deflection = +9 mm, (**d**) x-axis beamwidth of different focus positions, and (**e**) y-axis beamwidth of different focus positions.

Figure 14 shows the transverse distance of focus controlled on the y-axis by beam deflection, and the offset distance is 9 mm. Figure 14a–c shows the focused sound field with a focus offset ±9 mm from the transverse y-axis wheelbase based on the position 27 mm below the skull. Figure 14d,e show the width of the x-axis and y-axis of the focus beam, respectively.

We use delay setting to make the transducer array achieve beam deflection focusing. In deflection focusing, focal points with a deflection distance of ±9 mm all form effective focusing. The peak sound pressure of focal points at different deflection distances is close, which indicates that focal points in transcranial phased array focusing are controllable.

### 4.5. Effect of Amplitude Regulation

Due to the differentiation of trabecular bone structures in different positions of the skull, the attenuation of ultrasonic waves transmitting through the skull varies. Therefore, the path of phased array focusing can be optimized by amplitude weighting the transducer array elements. The amplitude-weighted values of each array element can be obtained from the cross-correlation results calculated in Equation (10). The amplitude-weighted value $A_{j}$ of jth element is calculated as [58]:

(11) $$A_{j}=\frac{N\cdot \max(R_{j}(\tau ))}{\sum_{j=1}^{N}\max(R_{j}(\tau ))},N=128$$

**Figure 15:**
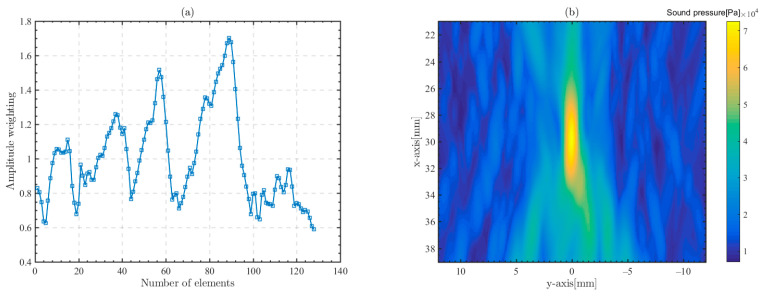
Amplitude weighted for each array element and the focused sound field after amplitude modulation, with the origin of the plot located 50 mm below the center array element of the transducer array. The frequency of the focused ultrasonic waves is 1 MHz. (**a**) Amplitude weighting of each array element; (**b**) focused ultrasonic field after amplitude modulation.

N is the number of array elements and the amplitude modulation is the initial amplitude of the jth element multiplied by the amplitude weighting $A_{j}$. Figure 15a shows the amplitude regulation value of each array element. Similarly, we set the focal point at 30 mm below the skull, and 50 mm below the center array element of the transducer array. The frequency of the focused ultrasonic waves is 1 MHz. The peak amplitude of the sound pressure at the focal point is 7.26 × 10^4^ Pa, as shown in Figure 15b. It can be found that the peak amplitude of the sound pressure at the focal point in Figure 11a,c is 8.39 × 10^5^ Pa and 6.71 × 10^4^ Pa, respectively. Comparing Figure 11a,c and Figure 15b, it can be concluded that the peak amplitude of the sound pressure at the focal point is attenuated by $20\mathrm{lg}\left(\frac{8.39\times 10^{5}}{6.71\times 10^{4}}\right)=21.9\mathrm{dB}$ and $20\mathrm{lg}\left(\frac{8.39\times 10^{5}}{7.26\times 10^{4}}\right)=21.2\mathrm{dB}$ without and with amplitude modulation. Therefore, the amplitude modulation method can increase the focusing effect and the sound pressure peak amplitude improved by 0.7 dB, i.e., the sound pressure amplitude is increased by $\frac{7.26\times 10^{4}-6.71\times 10^{4}}{6.71\times 10^{4}}=8.2\%$.

The amplitude regulation of the transducer array can improve the focusing efficiency to some extent. Amplitude regulation actually adjusts the transducer’s contribution to the focusing point according to the merit of the focusing path, which tends to be related to the porosity in the bone trabeculae and the complexity of the bone structure. 

## 5. Preliminary Experimental Verification

**Figure 16:**
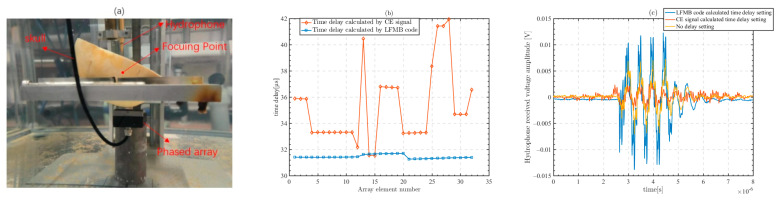
Using the time delay setting of the 32-element phased array for focusing. The hydrophone receives signals transmitted through the skull on the other side of the skull at the focusing point, and the received signals are displayed in an oscilloscope. (**a**) Positions of the line phased array, skull, focusing point and hydrophone, with the focusing point 50 mm directly below the center array element of the line array. (**b**) Time delay calculated by micro-CT simulation model. (**c**) Hydrophone received signal shown on an oscilloscope.

To validate our methodology and conclusions, we prepare the skull and phased array for preliminary experimental validation. Initially, we use micro-CT images to locate the position of micro-CT model slices. The CT data are imported into the Mimics software 21.0. We determine the number of CT slices corresponding to the specific structures (holes and gaps) of the skull based on the CT images. The location of the CT slices in the actual skull is determined by calculating the distance among the CT slices. The position of the acoustic window is similarly determined based on the above method. The transducer array is placed at the position set by the micro-CT model, and the transducer array is symmetric about the acoustic window. The center of the acoustic window on the skull is marked to confirm the position of the central element of the array. The skull is first placed close to the center element of the transducer and the relative distance between the array and the skull is ensured by measuring the movement of the platform. By adjusting the tilt angle and position of the skull to ensure that the relative positions of the acoustic window, the skull and the array are consistent with those in the micro-CT model. In the experiment, we use a 32-element line phased array with a wafer size of 1.5 mm × 20 mm, array spacing of 1.5 mm, and center frequency of 2 MHz. The focusing point is 50 mm away from the center array. A hydrophone is placed at the position of the focusing point. Prior to the experiment, the skull needs to be placed in water for 2 days to ensure that the pores were filled with water. The experiment is performed in the water tank with the water inside after more than 2 weeks of resting, as shown in Figure 16a.

We locate the position of the simulated computational acoustic window in the actual skull based on micro-CT image slices. Simulation calculations are set up based on the phased array, skull, and the distance to the focusing point. The CE signal and the LFMB code are used in the simulation to calculate the time delay from each array element of the transducer to the focusing point. The time delay calculated in the simulation model is the absolute delay from the transducer array element to the focusing point. The time delays of each array element of the transducer to the focusing point calculated in the simulation are shown in Figure 16b.

In the experiment, we use the time delay obtained from the simulation calculation for transcranial ultrasound focusing, and use the time delay calculated by the LFMB code, the time delay calculated by the CE signal, and the no time delay setting to excite the phased array, respectively. The transmit signal used for transcranial ultrasound focusing in the experiment is a 4-cycle 2 MHz frequency sine wave, and the signal received by the hydrophone at the focusing point is shown in Figure 16c. It can be seen that the waveforms of the signals received by the hydrophone are all distorted and scattered. The peak value of the focusing voltage is 11.2 mV with the time delay calculated using the LFMB code, 2.7 mV with the time delay calculated using the CE signal, and 7.4 mV with no time delay. The strong distortion of the transcranial CE signal leads to errors in the time delay calculation and reduces the received signal voltage at the focusing point, whereas the LFMB code improves the accuracy of the time delay calculation, so the received signal voltage at the focusing point is 8.5 mV higher than that with time delay setting calculated from the CE signal and 3.8 mV higher than that with no delay setting. 

The above experimental results indicate that the method proposed in this paper is effective and feasible.

## 6. Conclusions

In this paper, we use micro-computed tomography to obtain micro-CT images with a resolution of 60 µm. Based on these images, we construct the micro-CT model, which can restore the pore and bone layer structure of trabecular bone. Model calculations verify that the scattering of ultrasonic waves by trabecular bone pores leads to the high attenuation characteristics of the skull. Moreover, ultrasonic waves transmitting through the skull produce frequency shifts and scattering superimpositions.

Therefore, we adopt the pulse compression method and design the LFMB code to refine the calculation of the time delay. The combination of the pulse compression method and the cross-correlation calculation can effectively improve the accuracy of the time delay acquisition of the complex received signal. Using the time delay obtained by this method, the acoustic field characteristics of phased array focusing are investigated. We evaluate the effects of the signal frequency, beam deflection, and amplitude modulation on transcranial phased array focusing.

In previous studies, the skull is considered to be a high attenuation medium layer with a positive frequency correlation. Our research by the micro-CT model shows that the skeleton medium does not show high attenuation, but the pore and bone layer structure of trabecular bone greatly attenuates the ultrasonic waves transmitted through the skull. The attenuation caused by scattering is further enhanced with the increase in the frequency of focused ultrasonic waves. The trabecular bone structure of the skull varies greatly in different locations, which indicates that transcranial ultrasonic focusing can improve focusing efficiency by using regulatory methods. Amplitude regulation is a simple method to optimize focusing efficiency, which optimizes the ultrasound focusing path by weighting the amplitude of the array elements. Meanwhile, we investigated the feasibility of beam deflection focusing of linear arrays by time delay setting. With accurate time delay calculated from the LFMB code, the transducer array can be focused at different positions and depths in the skull. 

In the implementation of transcranial ultrasound focused therapy using the phase aberration correction method, the skull is simply considered as a nonuniform triple media layer, and in this idealization, the fine structure of the trabecular bone (nonuniform media) is reduced to a uniform medium, where refraction-induced intracranial light ray path deflections are ignored. This leads to a computationally incorrect estimation of the focused acoustic field as well as an actual focal shift. The cranial microstructural model developed in this paper helps to analyze the unpredictable wave-trajectory bending induced by the trabecular layer, thus providing further insights into the main factors contributing to transcranial ultrasound attenuation as well as beam scattering and deflection. In addition, the pulse compression method is universally applicable to all transcranial ultrasound computational models, and works well to improve accuracy in all time-delay calculations in complex media. The pulse compression method provides a reference for fast and accurate phase estimation for more precise transcranial ultrasound focusing and treatment. The preliminary experiments have been validated in this paper and we will further improve the experiment and demonstrate the availability of the method in transcranial ultrasound focusing.

## Data Availability

The data presented in this study are available on request from the corresponding author. The data are not publicly available due to confidentiality.
